# Single cell multi-omics of fibrotic kidney reveal epigenetic regulation of antioxidation and apoptosis within proximal tubule

**DOI:** 10.1007/s00018-024-05118-1

**Published:** 2024-01-25

**Authors:** Zhejun Chen, Liqing Ye, Minyan Zhu, Cong Xia, Junfen Fan, Hongbo Chen, Zhijian Li, Shan Mou

**Affiliations:** 1 Department of Nephrology, The First Affiliated Hospital of Zhejiang Chinese Medical University (Zhejiang Provincial Hospital of Chinese Medicine), Hangzhou, 310000 Zhejiang China; 2 Department of Nephrology, Molecular Cell Lab for Kidney Disease, Renji Hospital, School of Medicine, Shanghai Jiao Tong University, No 1630, Dong Fang Road, Shanghai, 200127 China; 3 Broad Institute of Harvard and MIT, Cambridge, MA 02142 USA

**Keywords:** Multimodal, scRNA-seq, Fibrotic kidney, Antioxidation, snATAC

## Abstract

**Background:**

Until now, there has been no particularly effective treatment for chronic kidney disease (CKD). Fibrosis is a common pathological change that exist in CKD.

**Methods:**

To better understand the transcriptional dynamics in fibrotic kidney, we make use of single-nucleus assay for transposase-accessible chromatin sequencing (snATAC-seq) and single-cell RNA sequencing (scRNA-seq) from GEO datasets and perform scRNA-seq of human biopsy to seek possible transcription factors (TFs) regulating target genes in the progress of kidney fibrosis across mouse and human kidneys.

**Results:**

Our analysis has displayed chromatin accessibility, gene expression pattern and cell–cell communications at single-cell level in kidneys suffering from unilateral ureteral obstruction (UUO) or chronic interstitial nephritis (CIN). Using multimodal data, there exists epigenetic regulation producing less Sod1 and Sod2 mRNA within the proximal tubule which is hard to withstand oxidative stress during fibrosis. Meanwhile, a transcription factor Nfix promoting the apoptosis-related gene Ifi27 expression found by multimodal data was validated by an in vitro study. And the gene Ifi27 upregulated by in situ AAV injection within the kidney cortex aggravates kidney fibrosis.

**Conclusions:**

In conclusion, as we know oxidation and apoptosis are traumatic factors during fibrosis, thus enhancing antioxidation and inhibiting the Nfix-Ifi27 pathway to inhibit apoptosis could be a potential treatment for kidney fibrosis.

**Supplementary Information:**

The online version contains supplementary material available at 10.1007/s00018-024-05118-1.

## Introduction

It has been reported that almost 700 million patients suffer from chronic kidney disease (CKD) worldwide, without proper treatment, CKD will finally progress to end-stage renal disease(ESRD) [1], which requires renal replacement treatment. It brings great economic pressure and results in low quality of life for patients. Until now, there has been no particularly effective treatment for CKD. Therefore, to develop new therapeutic methods for CKD, we try to understand the pathological mechanism of CKD. As we know, Fibrosis is a common pathological change that exists in CKD from a variety of etiology [2], but how fibrosis forms remains undiscovered. It is known that fibrotic kidneys and CKD can caused by maladaptive repair of renal tubular epithelial cell [3]. However, the mechanism underlying the maladaptive repair promoting kidney fibrosis remains unclear. Hence, it is urgent to explore the mechanisms of maladaptive repair leading to fibrosis.

A mouse model called unilateral ureteral obstruction (UUO) is a classic model to study renal tubulointerstitial fibrosis. During UUO, besides tubular damage, oxidative stress, inflammation, lipid metabolic disturbance, and mitochondrial dysfunction all contribute to fibrotic progression [4]. To have a better understanding of a variety of mechanisms involved in kidney fibrosis, single-cell RNA sequencing (scRNA-seq), which has pushed our understanding of disease at a single-cell level, is a better tool for research [5]. And recently it has been extended to combine the epigenome with transcriptome at the single-cell resolution for understanding biological processes at the level of transcriptional regulation. The single-nucleus assay for transposase-accessible chromatin sequencing (snATAC-seq) approach makes use of hyperactive Tn5 transposase to locate accessible chromatin at single-cell resolution [6], then besides providing complementary information to scRNA-seq through predicting transcription factors(TFs) activity, the snATAC-seq datasets can also forecast cis-regulatory DNA networks [7–9]. To better understand the transcriptional dynamics in fibrotic kidney, we make use of snATAC-seq and scRNA-seq from GEO datasets and perform scRNA-seq of human biopsy to seek possible TFs regulating target genes in the progress of kidney fibrosis across mouse and human kidneys.

## Methods

### Geo datasets processing

The downloaded SRA files, acquired by SRA-Toolkit, were split into fastq files, then fastq files with proper name entered into the cellranger count process, then the output file can be used for further downstream analysis.

### Ethics approval and consent to participate

The patient described in this study consented to the ethics committee review of Renji Hospital affiliated to Shanghai Jiao Tong University School of Medicine. Samples were obtained under local ethical approval. And the committee’s reference number is [2018] 049.

### Human kidney samples for scRNA-seq

Four kidney biopsies were used for scRNA-seq. Two transplantation donor kidneys were used as normal donor while two patients were used as Chronic interstitial nephritis (CIN) comfirmed by histopathology. One donor sample was confirmed as IgA deposition, whereas another donor sample was relatively normal besides reperfusion injury. The patient described in this study consented to the ethics committee review of Renji Hospital affiliated to Shanghai Jiao Tong University School of Medicine. Samples were obtained under local ethical approval. Samples were prepared according to 10X Genomics Single Cell 3’ v2 or v3 Reagent kit user guide.

### Tissue processing and single-cell dissociation

The fresh renal biopsy was preserved in cold PBS(1X), then we minced the biopsy into small pieces with a razor blade and incubated the pieces immersed in freshly prepared dissociation buffer containing enzymes from Multi Tissue Dissociation Kit (Miltenyi Biotec) at 37 °C. Dissociated cells were harvested every 10 min by passing the cell suspension through a 70-µm cell strainer (FALCON) into 10% FBS buffer on ice. This digestion procedure was repeated three times until most of the tissue had been dissociated into single cells. Finally, we collected cells by centrifugation at 1500 rpm for 5 min, resuspended cells in PBS(1X) containing 5% FBS and the resuspended cells were passed through a 40-µm cell strainer (FALCON) to further remove cell clumps and large fragments. Cell viability was approximately 85% as assessed by Trypan Blue staining for further constructing 10X microreaction system.

### ScRNA-seq data processing

We use Cell Ranger count to deal with the fastq files generated from 10X Genomics 3′ sequencing, the main output files of Cell Ranger count are gene-cell count matrices. Then we make use of R package SoupX to correct ambient RNA [10], then the cells, which were less than 50% mitochondria transcripts, with more than 200 and less than 3000 genes were retained. According to the instructions of SATIJALAB (https://satijalab.org/seurat/) [11], each dataset were integrated with batch effect corrected by R package Harmony [12], then the standard process of the calculation of 2000 high-variance genes, data normalization, scaling, dimensional reduction, clustering was performed by constructing a KNN graph and applying the Louvain algorithm. We employ UMAP to present dimensional reduction and we annotate each cell clusters based on the expression of known specific markers [11], and differentially expressed genes within one cell type across disease and con samples were calculated with the Seurat FindMarkers function. We define adjusted *p*-values < 0.05 as significance. And Gene expressions were visualized with FeaturePlot, VlnPlot or DotPlot function on Seurat.

### Ligand-receptor interaction found by CellChat

Ligand-receptor analysis was performed by CellChat according to instructions on GitHub (https://github.com/sqjin/CellChat) [13]. Briefly, the UUO or CIN single-cell data were extracted from the integrated scRNA-seq dataset, and the CellChat object was created using the create CellChat function. The created CellChat objects were then preprocessed by executing the process of identifyOverExpressedGenes, identifyOverExpressedInteractions and projectData functions, and communication probability and the cell–cell communication was computed and inferred with the default setting. The data were visualized with netVisual_heatmap, netAnalysis_dot and so on.

### SnATAC-seq data processing

We make use of Cell Ranger ATAC (v. 1.1.0) count to align the fastq files to the mm10(GRCm38) reference. Then R packages ArchR [14] was used to only keep the nucleus with a number of fragments more than 1000 and TSS enrichment more than 4. Similarly, we integrated datasets by using Harmony. Then UMAP was used to construct the K-nearest neighbour graph to visualise the data. And Cell types were annotated by transferring cell type identities of the scRNA-seq data to the snATAC-seq data, and as the insertion sequence bias of the Tn5 transposase, bias-corrected deviations with chromVAR for each sample were also calculated. TFs footprinting was performed using the ArchR getFootprints() function. Then to find possible enhancer sites, co-accessibility was done to predict regulatory interactions. And the H3k27ac CHIP-seq data visualized by IGV was also used to evidence the enhancer sites.

### TFs found by ArchR

To reproduce our peak calling done by Macs2, pseudo-bulk replicates were created, after identification of peaks, we found possible TF binding sites according to the most enriched motifs in those peaks by using R packages ArchR.

### The proteomics of kidney tissue during UUO, H3k27ac ChIP-seq of kidney tissue and spatial transcriptomics of sham and 6 w after ischemia–reperfusion injury(IRI) for integrative analysis

The Protein profiling by Mass Spec of kidney samples from different time points (before the obstruction, day 3, 7 and 14 after the obstruction) over the course of UUO was downloaded from GSE126182, the expression level of genes of interest was visualized by Graphprism. H3k27ac ChIP-seq of kidney tissue was downloaded from GSE114294, and the peak visualization was done by IGV software. The processed data of sham and IRI-6w from GSE182939 [15] was used to visualize spatially transcriptomics’ difference between normal and fibrosis.

### Plasmid construction

After RNA interference targets were designed, single-stranded DNA oligo-containing interference sequences was synthesized, and double-stranded DNA(dsDNA) was produced by annealing pairing. Then the dsDNA was directly connected to the BR-V108 vector through the cleavage sites at both ends. The connected products were transferred into the prepared Escherichia coli, and the positive recombinants were identified by PCR and sent for sequencing verification. Plasmid extraction was carried out for the correct clones. The RNA interference sequences were listed below, Human- NFIX-1,ATCAAAGAACTGGATCTTTATCT; Human-NFIX-2,CGGACAATCAGATAGTTCAAACC; Human-NFIX-3,AGGAGTTTGTGCAGTTTGTGTGC.

Restriction enzyme digestion was used to obtain the linearized LV-013 vector, and PCR amplification was performed to prepare the gene NFIX fragment, and the sequences of the 5′ and 3′ end of the amplification product are completely consistent with the sequences of the two ends of the linearized cloning vector, respectively. Linearized vector and target gene amplification products were used to prepare the reaction system, and the recombinant reaction was carried out to realize the in vitro recombination of linearized vector and target gene fragments. The recombinant plasmid was transformed, the monoclone on the plate was selected for PCR identification, and the positive plasmid was sequenced. High-purity plasmid was obtained by expanding the culture for subsequent experiments.

### RT-qPCR

HK-2 cells were cultured, and the constructed NFIX knockdown and overexpressed plasmid were transfected into HK-2 cells through lipo transfection system. The total RNA was extracted from the HK-2 cells by Trizol, For Reverse transcription, with all-in-One first-strand cDNA synthesis kit (Transgene), the total RNA of the 20 µL reaction system(containing 5*supermix 4 µL and Genomic DNA remover 1 µL) could be ≤ 1 µg. The volume was replenished to 20ul with rnase-free water. After gently mixing, the reaction was incubated at 42 °C for 15 min, and heated at 85 °C for 5 s. For realtime qPCR, generally, 3 multiple wells are needed. QPCR primers (10uM) are used to take 0.5 µL for each and pre-mix with 2* SYBE Green 5 µL, and the reaction system for each well is 10 uL. For data analysis, we calculated the relative expression of each gene based on the Ct value that came out and calculated the formula as 2^(internal reference Ct value—Ct value of the target gene). The qPCR primers are listed below (Table 1).

**Table 1:** The real time qPCR primers

Gene symbol	Upstream primer sequence	Downstream primer sequence
NFIX	CAGCAGTCGAGCCCGTAT	TCAGAAAGTTGCCGTCCC
IFI27	CTCTGGCTCTGCCGTAGTTT	ATGGAGGACGAGGCGATT
GAPDH	TGACTTCAACAGCGACACCCA	CACCCTGTTGCTGTAGCCAAA

### In situ injection of AAV(serotype 9) into kidney and UUO model

AAV-OE virus packaging plasmid was constructed first, and after purification and titration, AAV virus was injected into 3 independent sites (1*10^12^ copy number) through the kidney cortex to achieve overexpression of target molecules in kidney tissue [16]. After 3 weeks, the mouse was subjected to UUO operation [17] to induce kidney fibrosis, and kidneys were harvested at 7d after UUO establishment.

### Masson staining

Masson staining was performed according to manufactures’ instructions(Yeasen Biotechnology, 60532ES74).

### Western Blot and analysis

Tissues were lysed in a lysis buffer (Beyotime Biotechnology). Proteins were separated by SDS-PAGE according to molecular weight and transferred onto PVDF membranes. Membranes were blocked with 5% skim milk and were incubated with the appropriate primary antibodies diluted 1:1000 and then secondary antibodies conjugated with HRP. Protein bands were visualized by an ECL reagent. And Image-Pro Plus 6.0 software was used to select each strip and analyze the gray value, and the ratio of gray value between the target protein and the protein GAPDH was calculated.

### Statistical analysis

No statistical methods were used to predetermine the sample size. Experiments were not randomized and investigators were not blinded to allocation during library preparation, experiments or analysis. Statistical analysis was performed in GraphPad Prism and *P*-values were estimated by unpaired *t*-test.

## Results

### ScRNA-seq of fibrotic mouse and human kidney and snATAC-seq of fibrotic mouse kidney

**Fig. 1:**
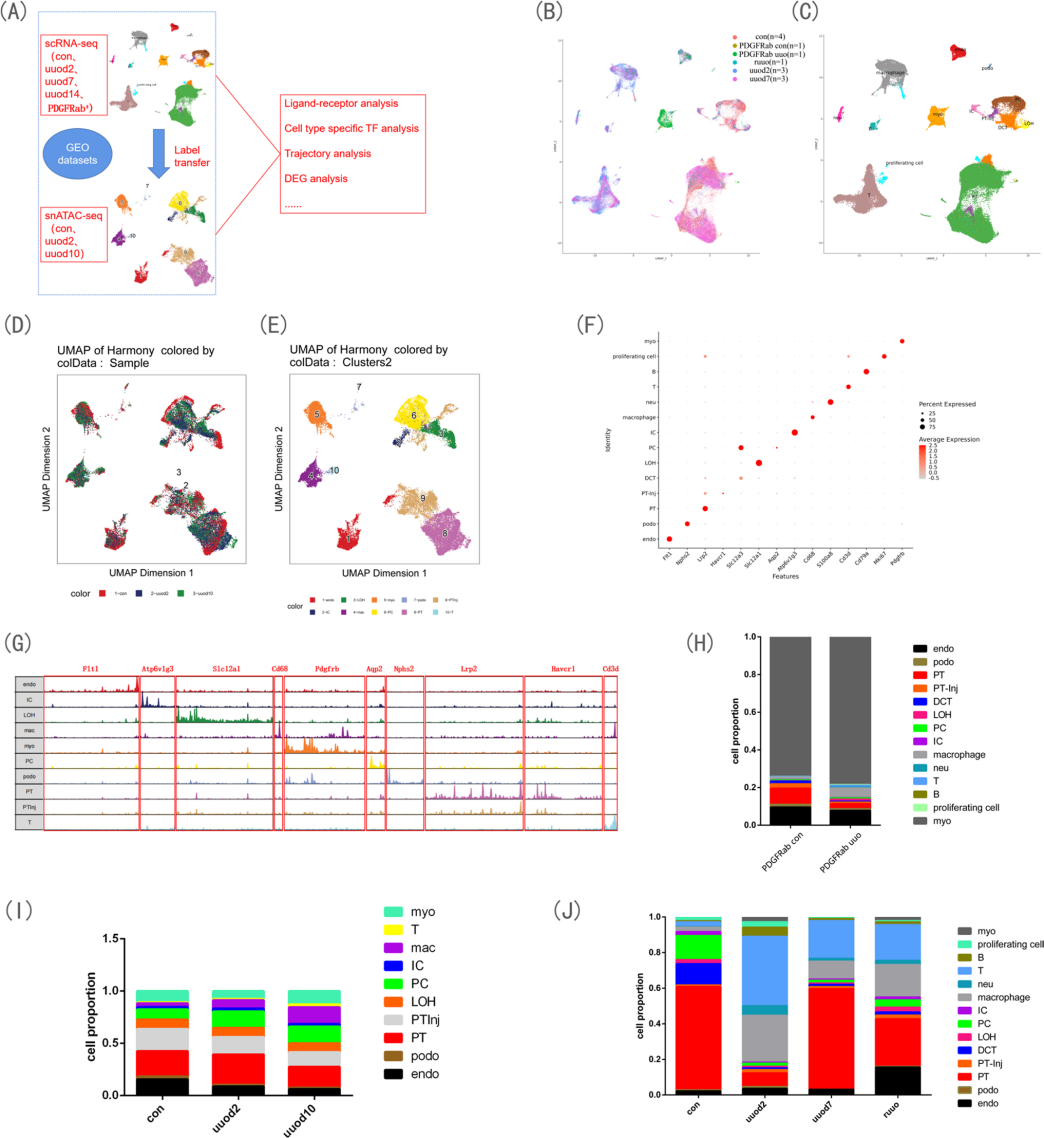
ScRNA-seq and snATAC-seq of fibrotic mouse kidney. **A** The work flow of integration of GEO datasets and cell type annotation transferred from scRNA-seq to snATAC-seq for further downstream multiomics analysis. **B** ScRNA-seq data, integrated from samples of con, uuod2, uuod7, ruuo and pdgfrab positive cells of con and uuo by harmony, were visualized as a UMAP plot. **C** ScRNA-seq data, annotated as endothelium(endo),podocyte(podo), proximal tubule(PT), PT-Injuried(PT-Inj), distal convoluted tubule(DCT), loop of henle(LOH), principal cell(PC), intercalated cell(IC), macrophage, neutrophil(neu), T, B, myofibroblast(myo), proliferating cell, were visualized as UMAP plot. **D** SnATAC-seq data, integrated from samples of con, uuod2, uuod10 by harmony, were visualized as UMAP plot. **E** SnATAC-seq data, annotated as endo, podo, PT, PTInj, LOH, PC, IC, macrophage(mac), T, myo, were visualized as a UMAP plot. **F **The markers used to annotate cell types of scRNA-seq data were visualized as DotPlot.** G** The markers used to annotate cell types of snATAC-seq data were visualized as Peak. **H** The cell proportion in PDGFRab positive cells of UUO sample and con sample from scRNA-seq data. **I** The cell proportion in con, uuod2 and uuod10 samples from snATAC-seq data. **J** The cell proportion in con, uuod2 and uuod7 and ruuo samples from scRNA-seq data

**Fig. 2:**
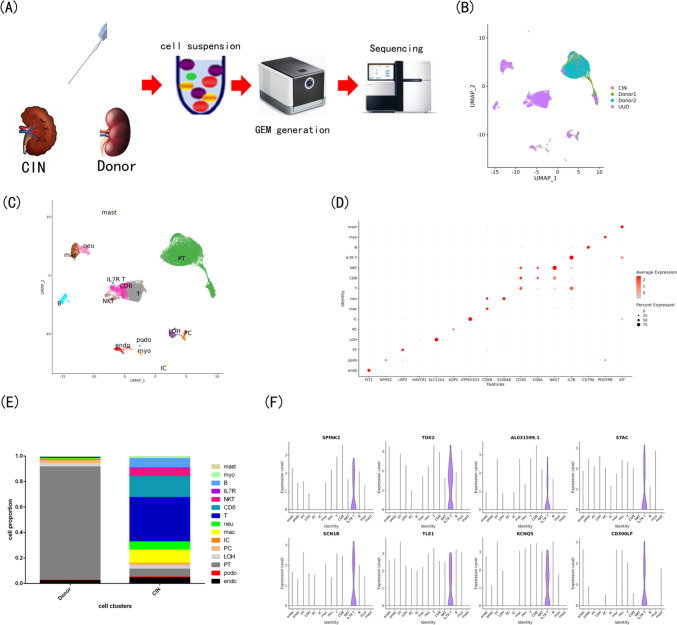
ScRNA-seq of fibrotic human kidney. **A** The workflow of single-cell RNA seq for human kidney biopsy sample.** B** ScRNA-seq data, integrated from samples of donor, CIN and UUO by harmony, were visualized as UMAP plot.** C** ScRNA-seq data, annotated as endo, podo, PT, LOH, PC, IC, mac, neu, mast, T, IL7R T, CD8, NKT, B, myo, were visualized as UMAP plot. **D** The markers used to annotate cell type of scRNA-seq data were visualized as DotPlot.** E** The cell proportion in Donor and CIN (samples of CIN and UUO were defined as CIN) samples from scRNA-seq data. **F** Interestingly, one cell type, expressing more IL7R, is specific to the CIN sample, and we use VlnPlot to show the typical markers of this cell type

To obtain the difference in kidney transcriptome between normal and fibrotic kidney, we acquire 100024 high-quality cells after quality control (Supplementary Fig. 1A) which form 14 clusters through integrating GEO datasets [2, 18–20] (GSE197266, GSE140023, GSE145170 and GSE182256). They are endothelium(endo), podocyte(podo), proximal tubule(PT), PT-Injuried(PT-Inj), distal convoluted tubule(DCT), loop of henle(LOH), principal cell(PC), intercalated cell(IC), macrophage(mac), neutrophil(neu), T, B, myofibroblast(myo), proliferating cell (Fig. 1B and C). While to analyze the mechanisms underlying the change of transcript during the fibrosis process, we also make use of GEO data [21] (GSE139950) to finally acquire 33379 high-quality nucleus, and we transfer the cell labels of scRNA-seq data to snATAC-seq data, finally we obtain 10 clusters, they are endo, podo, PT, PTInj, LOH, PC, IC, mac, T, myo (Fig. 1D and E). The markers to define the clusters of scRNA-seq were showed in DotPlot (Fig. 1F), while the gene peaks to define the clusters of snATAC-seq were shown in Fig. 1G. In Fig. 1H, we can see the increased cell proportion of myo and decreased cell proportion of PT in PDGFRab double positive cell of UUO sample compared to that of the con sample. While in the total kidney, a decreased cell proportion of PT was observed in the uuod2 kidney sample comparing with the con sample and an increased cell proportion of T and macrophage was found in the uuo group compared with the con group (Fig. 1J). In the snATAC assay, an increased cell proportion of T and macrophage was also found in the uuo group comparing with the con group (Fig. 1I). To have some implications of translational medicine, we acquired human kidney biopsy which is composed of two transplantation donor, one CIN and one UUO(which is caused by ureteral tumor, and the pathology was also CIN) (Fig. 2A and B). At last, we got 24103 high-quality cells after quality control (Supplementary Fig. 1B) which form 15 clusters. They are endo, podo, PT, LOH, PC, IC, mac, neu, mast, T, IL7R T, CD8, NKT, B, myo (Fig. 2C). The markers to define the clusters of scRNA-seq was shown in DotPlot (Fig. 2D), In Fig. 2E, we can see obvious decreased cell proportion of PT and increased cell proportion of immune cells in the CIN group comparing with the donor group. And IL7R T was only found in the CIN group (Fig. 2E). The specific markers to define the IL7R T cell cluster were showed by VlnPlot (Fig. 2F).

### UUO-related TF regulating Sod1 within PT was found by snATAC-seq

**Fig. 3:**
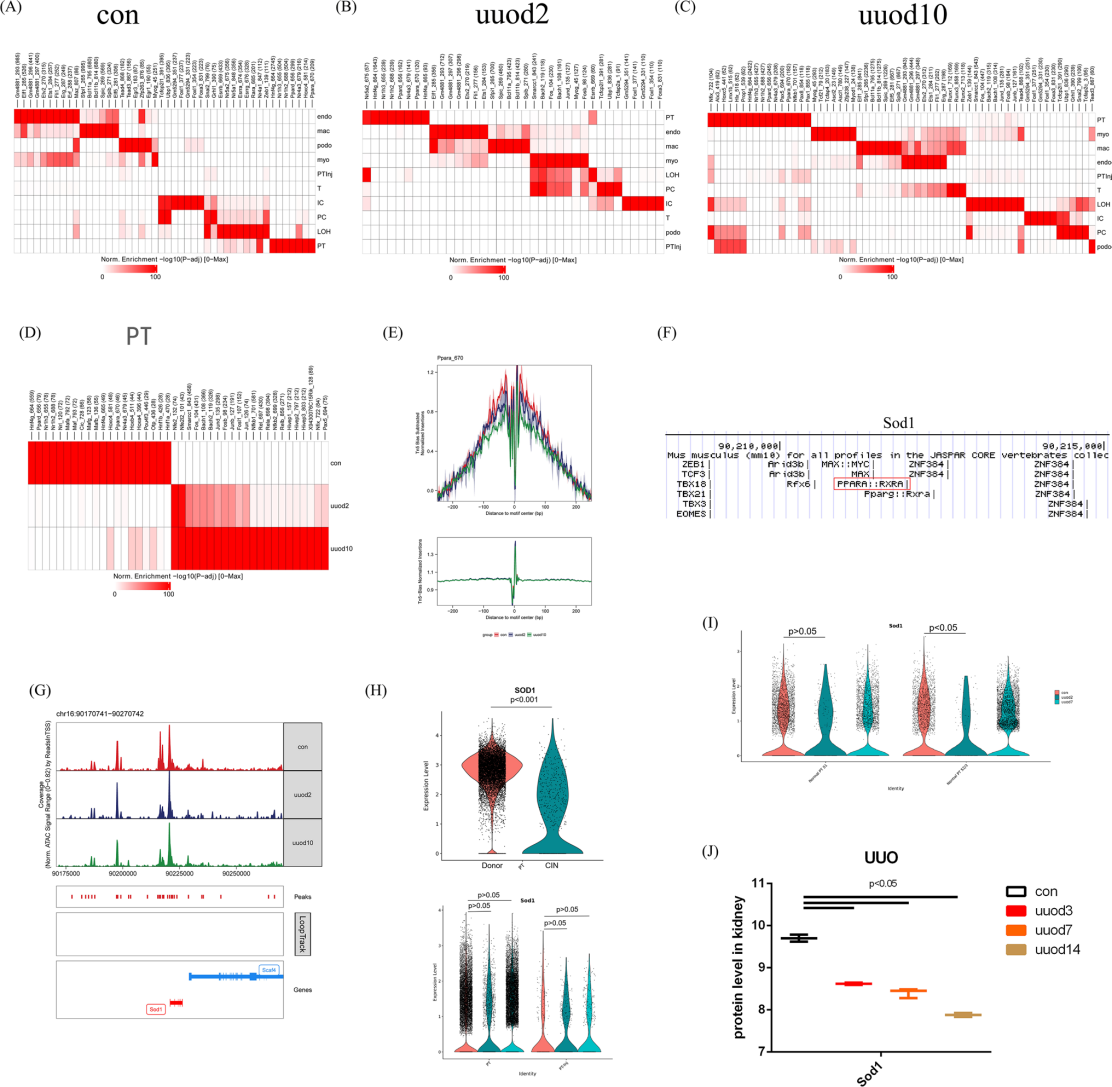
The lower binding activity of Ppara to the promoter of Sod1. The motif enrichment across all cell types in con (**A**), uuod2 (**B**) and uuod10 (**C**) were shown by Heatmap. **D** The motif enrichment within PT among con**,** uuod2 and uuod10 were shown by Heatmap.** E** The Ppara footprinting within PT among con, uuod2 and uuod10 were shown. **F** The TFBS predictions of Sod1 based on the JASPAR CORE vertebrates collection (2022) were shown, and we can see the binding site of Ppara was between chr16:90210000–90215000. **G** The lower accessibility marked by a peak around Sod1 promoter region in uuo sample comparing with con sample was found in the vicinity of chr16:90212500. **H** The Sod1 expression between fibrotic and con kidney was shown by VlnPlot. **I** The Sod1 expression within subclusters of PT between fibrotic and con kidney was shown by VlnPlot. **J** The protein level of Sod1 between the uuo kidney and the con kidney were shown

To seek the fibrosis stage-related TFs, we plot the cell type-specific TFs in a heatmap of con (Fig. 3A), uuod2 (Fig. 3B) and uuod10 (Fig. 3C). The representative TFs within PT across con, uuod2 and uuod10 were shown in Fig. 3D, then we search the a transcriptional regulatory network database named TRRUST (http://www.grnpedia.org/trrust) and find Ppars may regulate Sods, and the Ppara’ footprints within PT was showed in Fig. 3E, the TFBS predictions of Sod1 based on the JASPAR CORE vertebrates collection (2022) were showed in Fig. 3F, and we can see the binding site of Ppara was between chr16:90210000–90215000. The lower accessibility peak of Sod1 in the uuo sample comparing with con sample was found around chr16:90212500 (Fig. 3G). By using VlnPlot, the Sod1 expression between fibrotic and con kidney was shown, and compared with the donor sample, we can see an obvious decrease in Sod1 mRNA level within PT of the CIN sample (Fig. 3H), while in mouse kidney, the decreased Sod1 mRNA level can be found in PT subclusters (PT S2/3) of uuo comparing with those of con (Fig. 3I). In Fig. 3J, similar to the Sod1 mRNA level, the protein level of Sod1 in the uuo kidney was less than that in the con kidney.

### Coaccessibility network around the Sod2 locus

**Fig. 4:**
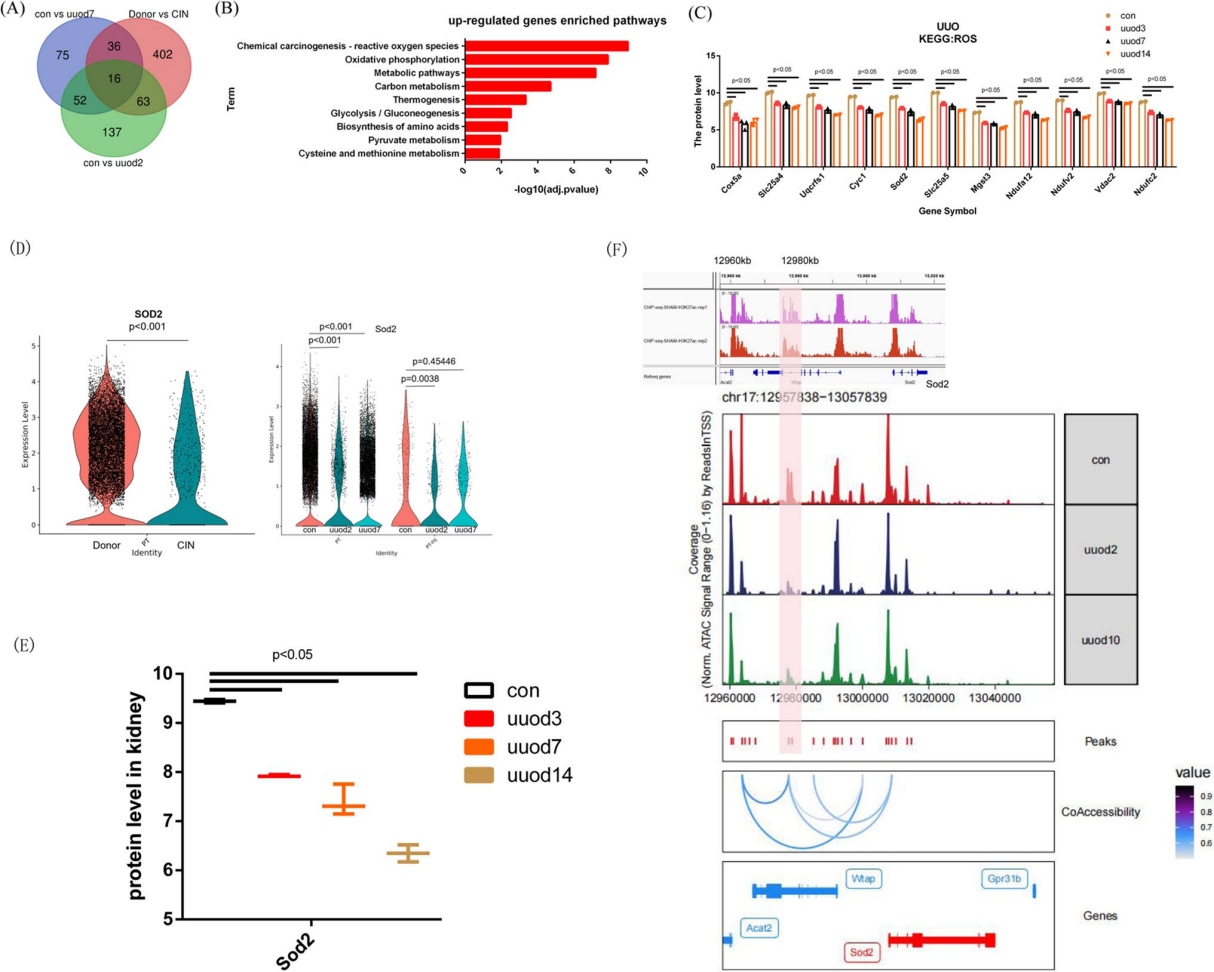
Coaccessibility network around the Sod2 locus. **A** The Venn diagram showed the common up-regulated genes within PT between the con vs fibrotic kidney. **B** The signaling pathway enriched by the common up-regulated genes of con vs uuod7 and Donor vs CIN were shown by a bar plot. **C** The protein level of genes, which are enriched in ROS signaling shown in Fig. 5B, between uuo kidney and con kidney were showed. **D** The Sod2 expression between fibrotic and con kidney was shown by VlnPlot. **E** The protein level of Sod2 between the uuo kidney and the con kidney were shown. **F** The ChIP-seq data of normal kidneys targeting H3k27ac were shown, and a peak appeared on chr17:12,970–12,980 kb. The co-accessibility network of Sod2 was shown by ArchR analysis, while a peak in chr17:12,970–12,980 kb seems to have an interaction with the promoter region of Sod2, and all these interactive peaks were lower in the uuo sample compared with the con sample

To find a common mechanism underlying the fibrosis development, the Venn diagram was used to show the common up-regulated genes within PT between con vs fibrotic kidney (Fig. 4A). We also use a bar plot to show the signaling pathway enriched by the common up-regulated genes of con vs uuod7 and Donor vs CIN, and the top signaling pathways ranked by −log10(adj.pvalue) was ROS signaling (Fig. 4B), and we show the protein level of genes from proteomics of GEO datasets, and all these genes enriched in ROS signaling were decreased in uuo kidney compared with con kidney (Fig. 4C). And we thought fibrosis development may be due to deficiency of Sod2 in uuo compared with con. Meanwhile, we checked the mRNA and protein level of Sod2, the lower Sod2 mRNA level within PT was found in CIN or UUO sample comparing with that in con kidney (Fig. 4D) and lower Sod2 protein level was also found in UUO kidney compared with con kidney (Fig. 4E). So we explore the epigenetic regulation of Sod2. The co-accessibility network of Sod2 was showed by ArchR analysis, while a peak in chr17:12970-12980 kb seems to have an interaction with the promoter region of Sod2, and all these interactive peaks was lower in uuo sample compared with con sample. Further analysis by the ChIP-seq data of kidney targeting H3k27ac were showed, and a peak appear on chr17:12970–12980 kb, so we thought the peak in chr17:12970–12980 kb may be a site of enhancer (Fig. 4F).

### The transcription factor Nfix regulate the Ifi27 expression which was involved in apoptosis and fibrosis

**Fig. 5:**
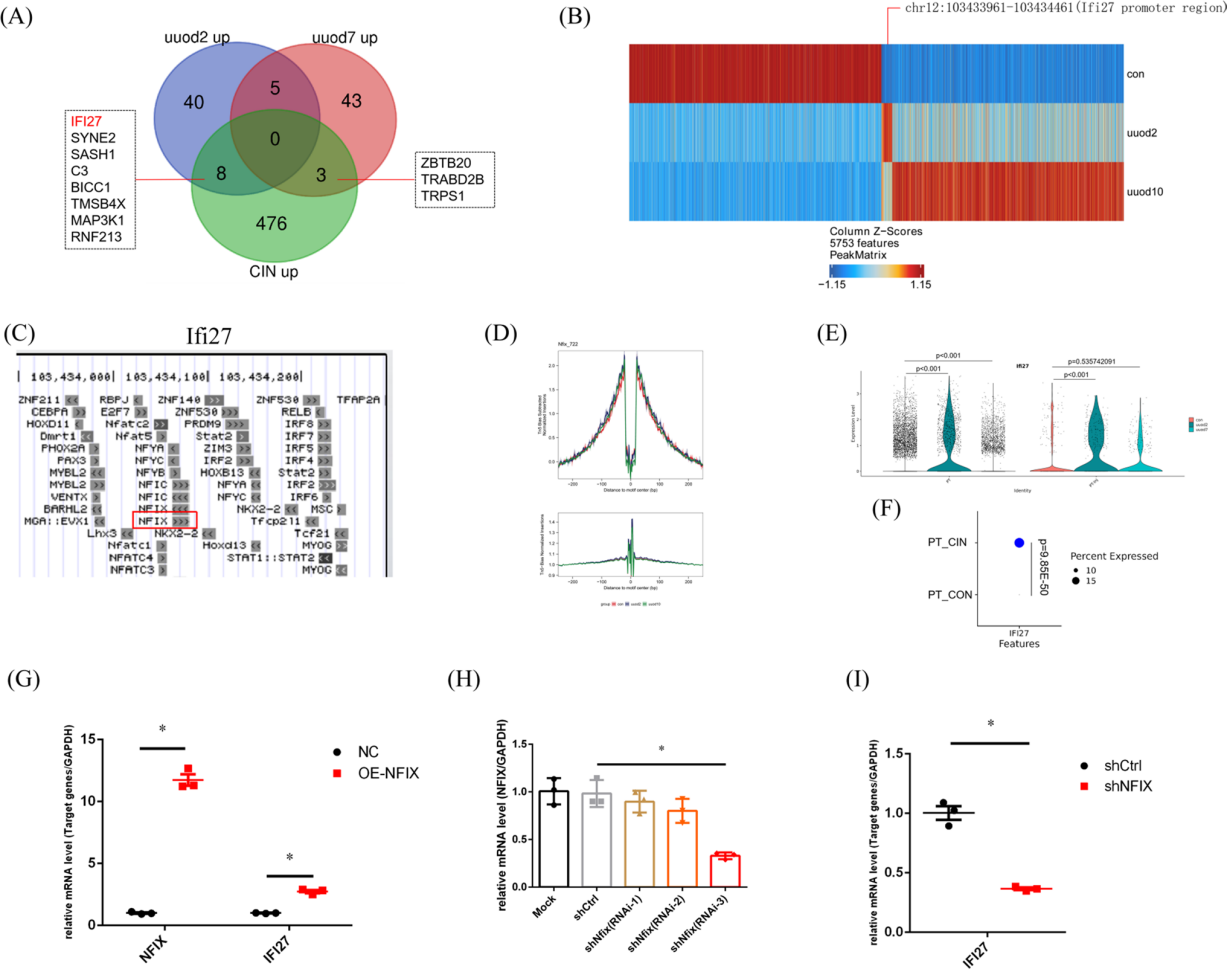
The transcription factor Nfix regulate the Ifi27 expression. **A** The Venn diagram was used to show the common up-regulated genes within PT between fibrotic kidney vs con, and 11 common genes among human and mouse samples were found respectively. The gene IFI27 was marked by the red color. **B** The differential peaks between the con and the uuo groups from snATAC data were used to compare with the promotor region of the 11 genes, and the differential peak chr12:103,433,961–103,434,461 was located in the promoter region of the gene Ifi27. **C** The TFBS predictions of Ifi27 based on the JASPAR CORE vertebrates collection,(2022) were shown, and the binding site of Ifi27 by NFIX was between chr12:103,434,000–103,434,100. **D** The Nfix’ footprints within PT were shown. **E** The Ifi27 expression between fibrotic and con kidney was shown using VlnPlot, and compared with the con sample, an obvious increase in Ifi27 mRNA level within PT was found in the uuod2 sample. **F** While in the human sample, an obvious increase in Ifi27 mRNA level within a fraction of PT was also found in CIN compared with that in the con kidney. The HK2 cell was transfected with the over-expression (**G**) and shRNA plasmid (**H**), and over-expression of NFIX can significantly promote the IFI27 expression (**G**), while knockdown NFIX by shRNA plasmid transfection can reduce the IFI27 expression (**I**). *p < 0.05

**Fig.6:**
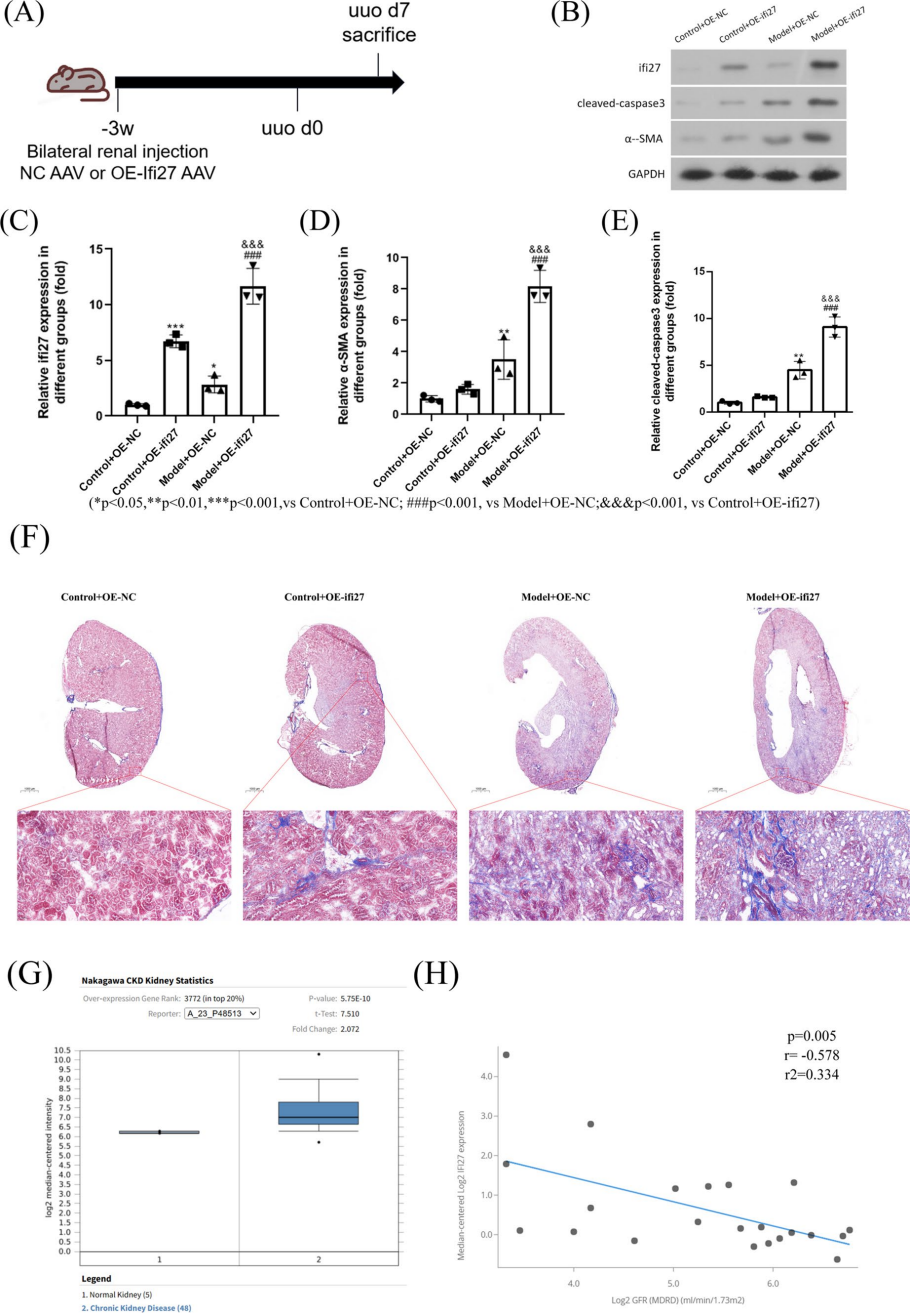
Ifi27 level within the kidney was associated with fibrosis. **A** The experimental flow chart of in situ AAV injection and UUO model. **B** The western blot was used to show the overexpression of Ifi27 can promote apoptosis and fibrosis. Then the statistical analysis of Ifi27 (**C**), α-SMA (**D**) and cleaved-caspase3 (**E**) protein level from immunoblotting was done. **F** The masson staining of kidney sections was used to show the general collagen level. **G** By searching the Nephroseq database, the Ifi27 was significantly increased in the CKD group compared with normal kidneys. And **H** a negative correlation between IFI27 expression and GFR(MDRD) was found in the tubule of con and diabetic kidney disease from the Nephroseq database

**Fig. 7:**
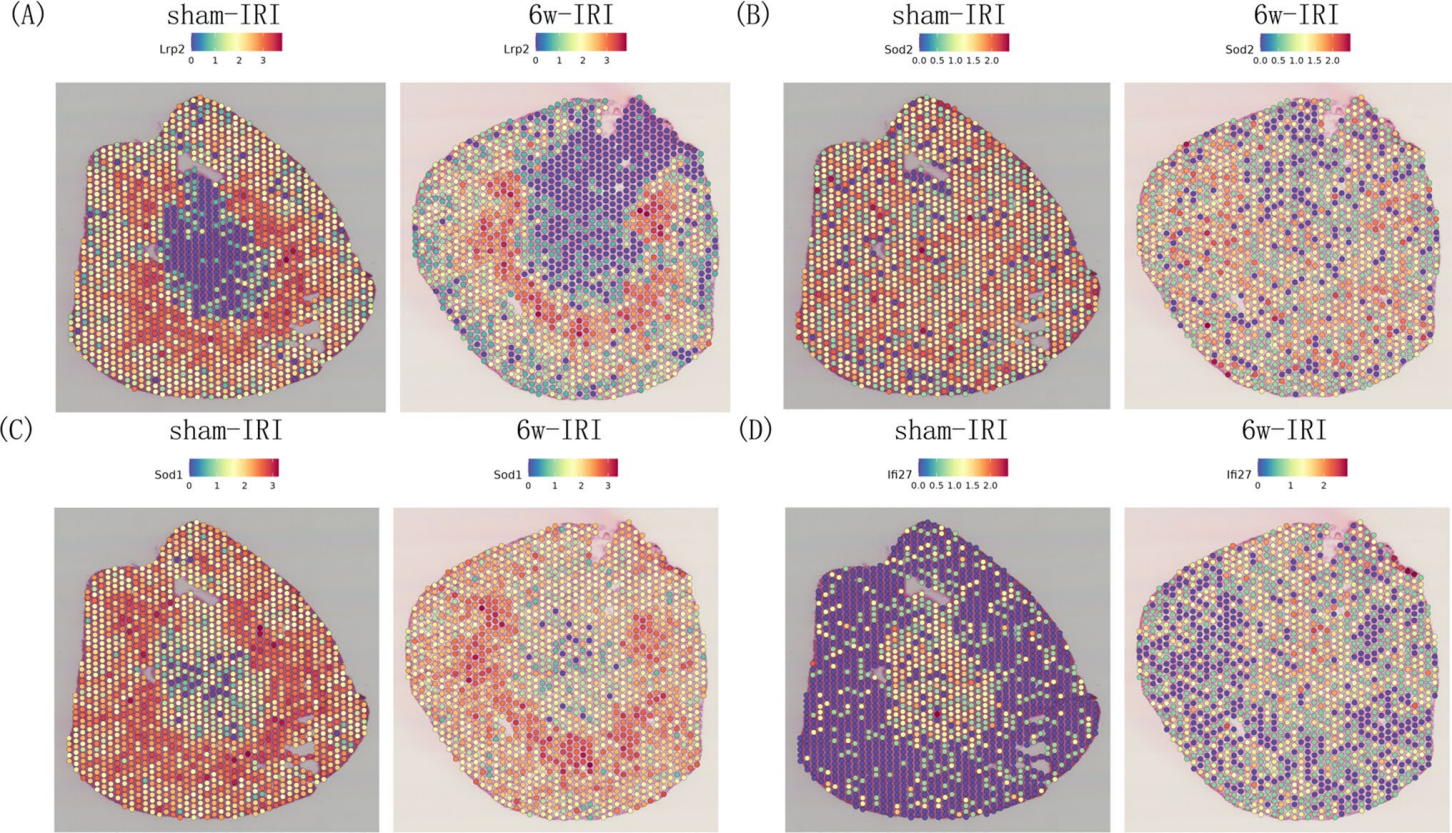
Spatial transcriptional level of Sod1, Sod2 and Ifi27 in the kidney of sham and 6w-IRI. We first used the gene Lrp2 to mark the PT (**A**), then the genes of concern including Sod2 (**B**), Sod1 (**C**) and Ifi27 (**D**) were showed spatially between sham-IRI and 6w-IRI

To find a common mechanism underlying the fibrosis development, the Venn diagram was used to show the common up-regulated genes within PT between fibrotic kidney vs con (Fig. 5A), and 11 common genes among human and mouse samples were found, respectively. The gene IFI27 was marked by the red color. Then we use the differential peaks between con and uuo groups from snATAC data to compare with the promotor region of the 11 genes. Then the differential peak chr12:103433961–103434461 was located in the promoter region of the gene Ifi27 (Fig. 5B). The TFBS predictions of Ifi27 based on the JASPAR CORE vertebrates collection (2022) were shown in Fig. 5C, and we can see the binding site of NFIX was between chr12:103434000–103434100. The Nfix’ footprints within PT was shown in Fig. 5D. By using VlnPlot, the Ifi27 expression between fibrotic and con kidney was shown, and compared with the con sample, we can see an obvious increase in Ifi27 mRNA level within PT of the uuod2 sample (Fig. 5E), which was consistent with the differential peak showed by heatmap (Fig. 5B). Meanwhile, an obvious increase in IFI27 mRNA level within a fraction of PT was found in CIN compared with that in the con kidney (Fig. 5F). Then to validate our analysis, we construct the over-expression(OE) and shRNA plasmid, after transfecting HK2 cell, OE NFIX can significantly promote the IFI27 expression (Fig. 5G), while knockdown NFIX by shRNA plasmid transfection can reduce the IFI27 expression (Fig. 5I). Then to explore the role of Ifi27 in kidney fibrosis, we designed an in situ AAV injection and UUO model, in detail, we have done a bilateral renal AAV injection to overexpress Ifi27, then uuo model was done 3 weeks later (Fig. 6A). At 7d after uuo establishment, then by using westernblot, the overexpression of Ifi27 within the kidney can promote the expression of cleaved-caspase3 and α-SMA under fibrosis conditions (Fig. 6B–E). Then the masson staining of kidney sections was used to show the general collagen level, the results also indicated that the Ifi27 overexpression in the kidney can promote the kidney fibrosis(Fig. 6F). In order to find the translational significance of Ifi27, by searching the Nephroseq database, we can find the Ifi27 was significantly increased in CKD group comparing with normal kidneys (Fig. 6G). And a negative correlation between IFI27 expression and GFR(MDRD) was found in the tubule of con and diabetic kidney disease from the Nephroseq database (Fig. 6H). To sum up, the level of apoptosis related gene Ifi27 within kidney regulated by Nfix was associated with kidney fibrosis. Meanwhile, to further validate the increased Ifi27 expression in intact kidneys, we use spatial transcriptomics data of sham and 6w after IRI, which we think it as a fibrotic kidney, from GEO datasets, Firstly, the gene Lrp2 was used to mark the PT (Fig. 7A), then the genes of concern including Sod2 (Fig. 7B), Sod1 (Fig. 7C) and Ifi27 (Fig. 7D) were showed spatially between sham-IRI and 6w-IRI. The Sod1 and Sod2 were significantly decreased in 6w-IRI kidney compared with those in sham-IRI kidney, while Ifi27 was obviously increased in the cortex region including PT cells at 6w after IRI, which was consistent with the single cell data.

## Discussion

Previous bulk-seq of kidney tissues is hard to distinguish cell-to-cell heterogeneity and identify the specific cell types in which the signaling pathways were activated or down-regulated. To overcome this limitation, we performed multimodal single-cell analysis on mouse uuo and adult human CIN kidneys to dissect the molecular difference along disease progression at single-cell resolution. Our analysis has displayed chromatin accessibility, gene expression pattern and cell–cell communications at single-cell level in UUO or CIN kidneys. In this paper, we combined as many datesets as possible to acquire relatively representative data, while we found the cell proportion of PT during the fibrotic process in different datasets seems to be heterogeneous. As the proportion of PT decrease in uuod2 or CIN, while the proportion of PT seems to increase in uuod7 compared with that in uuod2. ZM Chen et al. found a significant decrease in the PT cell ratio of uuod2 (6.15%) comparing with that of the control (70.94%) [17], which was consistent with our findings. And SR Cao et al. also found a decrease in the PT cell ratio of uuod3 (35.0%) compared with that of uuod1 (44.4%) [22]. While Conway et al. found the various kinds of PT proportion always decreased until 7d after uuo [19], which was controversial to the result of this paper. Because when we split all datasets included, we found the proportion of PT cells in uuod7 was similar to that in control from the datasets of Doke et al. [20]. Thus the cell ratio may have heterogeneity which was caused by experimental operation, individual differences in mice and so on. We believe single-cell studies with larger sample sizes can clarify this question in the future.

As the most abundant cell type, proximal tubule epithelial cells are also the most vulnerable to various pathogenic factors [2, 23], when PT suffers injury, PT initiates repair by dedifferentiation, when the damage persists, PT will undergo failed repair, this failed repaired PT has been identified by some markers [24], in this paper we also found the failed repair-PT (FR-PT) exists in uuo or CIN kidney (Supplementary Fig. 5). Previous studies found that overexpression of myo-inositol oxygenase (MIOX), a proximal tubular enzy me, can exacerbate kidney injury in cisplatin-induced acute kidney injury (AKI). which can be partially rescued in cisplatin-treated MIOX-KO mice [25]. While in the model of cadmium and gentamicin-induced kidney injury, overexpression of MIOX can also aggravate the phenotype of kidney injury through oxidative stress and necroptosis [26, 27], MIOX-transgenetic mice have accelerated tubulointerstitial fibrosis when they are challenged with diabetic kidney disease [28], in our datasets, a PT cluster expressing high Miox/MIOX was found across human and mouse (Supplementary Fig. 5), according to above research results, this PT cluster may be detrimental to the kidney. Previous study found FBP-1 activity in urine can be detected higher in children with idiopathic nephrotic syndrome (INS) than that of healthy children [29], in our study, we found the proportion of Fbp-1 high PT was higher in uuod2 comparing with con, while Fbp-1 high PT disappear in longer uuo time point (Supplementary Fig. 5). So FBP-1 may be a marker of early injury. As a protective role, Nicotinamide N-methyltransferase (NNMT) can reduce oxidative stress and cell death of PT induced by lipotoxicity [29], and NNMT can also improve renal fibrosis by targeting the TGF-β1 signalling pathway [30]. As NRF2-target marker genes [31], aldo–keto reductase 1C1(AKR1C1) may be a detoxifying molecule. And as our data show, a decrease in AKR1C1 hi-PT and NNMT hi-PT were found in CIN compared with the donor kidney (Supplementary Fig. 5).

In the part of cell–cell communication analysis, macrophage migration inhibitory factor (MIF) is a protective role involved in the process of kidney fibrosis. When mice were challenged with unilateral ureteral obstruction, genetic deletion or pharmacologic inhibition of MIF accentuated the fibrosis phenotype, while the fibrosis phenotype can be alleviated by the rescue assay. Furthermore, the MIF within the tubular cells rather than immune cells have protective effects, which was confirmed by the phenotype of Knockout mice [32]. In our data, we found Mif secreted from macrophage was activated in uuo sample (Supplementary Fig. 2), further experiments need to be done for the role of Mif within macrophage. In our data, the interaction of Ccl4-Ccr5 and Ccl6-Ccr2 within macrophage were activated in uuod2 and uuod7 samples, respectively, comparing with the con sample. And Ccl9 and Ccl6 secreted from macrophage respectively acting on Ccr1 of neu were activated in uuo sample comparing with con (Supplementary Fig. 2), thus suggesting innate immune activation existed in uuo, which is consistent with previous studies [33, 34].

As a main cellular source of ECM, Myofibroblasts contribute most to kidney fibrosis, by using genetically engineered mice to fate mapping the source of myofibroblasts in kidney fibrosis, the epithelial-to-mesenchymal transition(EMT) accounted for 5% of myofibroblasts during UUO development [35]. In this study, the alteration of TFs and variable genes during PT transition to myo could be used for further experimental verification (Supplementary Fig. 6), thus providing clues for reversing EMT during fibrosis.

Besides the above molecules involved in fibrosis, reactive oxygen species (ROS) overproduction induced by UUO can promote inflammation, oxidative stress, and apoptosis, which contributes to fibrosis [36, 37]. In this paper, we found a deficiency in the antioxidant capacity of UUO or CIN kidney, previous research found UUO induction resulted in impaired renal function along with the downregulation of antioxidant proteins, such as NRF2, NQO1 and SOD, as a result, more ROS produced finally leads to oxidative stress and fibrosis [38, 39]. While the expression of SOD2, an antioxidant enzyme, can protect against chronic kidney injury and renal fibrosis by reducing oxidative stress in renal tubular cells [40, 41]. Peroxisome proliferator-activated receptors (PPARs) are a kind of transcription factor having anti-inflammatory effects, one study found the activation of PPAR-α by PPAR agonists can improve UUO phenotype [42]. In this study, by searching the TRRUST database, Sod was one of the target genes of PPARs. Then we found motif footprints of PPAR-α, and less PPAR insertions of uuo group comparing with that of the con were found, which suggests less Sods expression in uuo group was due to the weak binding ability of PPAR to promoters of Sods. Our research also found high NR4A1 activities in the con kidney compared with uuo kidney in the snATAC-seq data. As we know, the nuclear receptor NR4A1, as an endogenous inhibitor of TGF-β signaling, can improve kidney fibrosis in mice [43], meanwhile, NR4A1 can upregulate the key enzymes of the TCA cycle which was found to be downregulated in uuo sample of this analysis [44].

H3k27ac ChIP-seq signal can be identified as an enhancer region [8], in this study, through combining H3k27ac ChIP-seq with snATAC coaccessibility analysis, we found a peak appearing on chr17:12970–12980kb may be a site of enhancer to regulate the Sod2’s promoter. Thus further gene editing assay need to be done for validation of the enhancer region.

Ifi27 was reported to be involved in apoptosis signaling, while the results seem to be somewhat controversial, IFI27 knockdown and over-expression in keratinocytes had no effect on apoptosis [45]. However, until now, the role of Ifi27 in kidney function has not been clarified. In this paper, we found the transcription factor Nfix can promote the Ifi27 transcription which may be associated with apoptosis and fibrosis.

## Conclusions

**Fig. 8:**
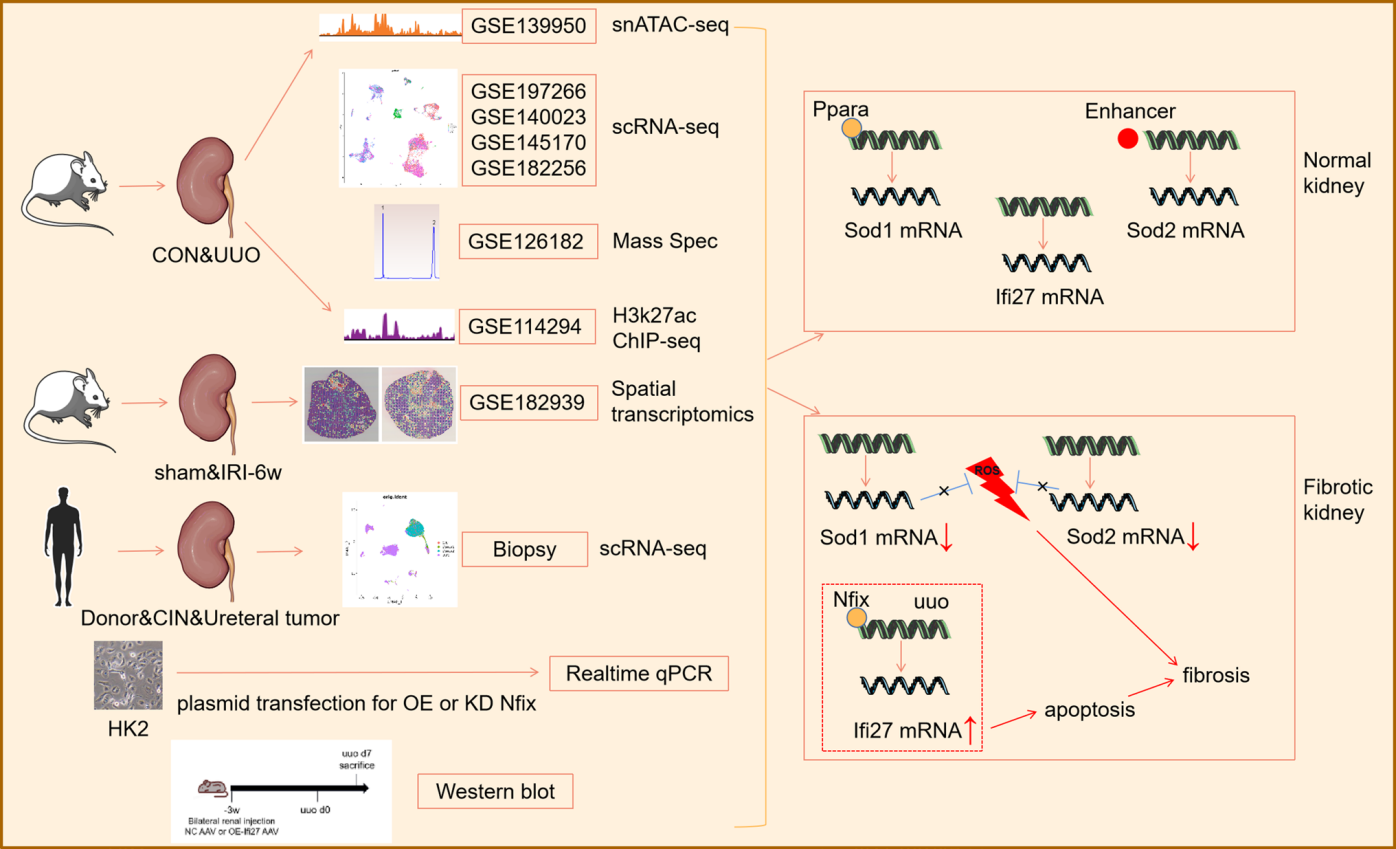
Summary of the general idea and main findings of this paper by using graphic abstract. In this paper, we make use of snATAC-seq and scRNA-seq of mouse kidney from GEO datasets and perform scRNA-seq of human biopsy to seek possible epigenetic regulatory mechanism in the progress of kidney fibrosis across mouse and human kidney. To evidence the analysis results, we also take advantage of proteomics of kidney tissue during UUO, H3k27ac ChIP-seq of normal kidney and spatial transcriptomics of mouse kidney at 6w after IRI. As a result, we found deficiency in epigenetic regulation of Sod1 and Sod2 within proximal tubule which is hard to withstand oxidative stress during fibrosis. Meanwhile, in vivo and in vitro experiments found that a transcription factor Nfix promoting the apoptosis-related gene Ifi27 expression involved in kidney fibrosis

Finally, we conclude that by using multimodal data, there exists epigenetic regulation leading to less Sod1 and Sod2 mRNA within the proximal tubule which is hard to anti-oxidative stress during fibrosis. Meanwhile, a transcription factor Nfix promoting the apoptosis-related gene Ifi27 expression was validated by an in vitro study. And the gene Ifi27 upregulated by in situ AAV injection within the kidney cortex aggravates kidney fibrosis. As oxidative stress and apoptosis are injurious phenotype, thus enhancing antioxidation and inhibiting the Nfix-Ifi27 pathway could be a potential treatment for kidney fibrosis (Fig. 8).

### Supplementary Information

Below is the link to the electronic supplementary material.

Supplementary file1 (DOCX 3332 KB)

## Data Availability

Besides the public GEO datasets(GSE197266, GSE140023, GSE145170 and GSE182256) for scRNA-seq, GSE139950 for snATAC-seq, GSE182939 for spatial transcriptomics, GSE126182 for proteomics and GSE114294 for H3k27ac ChIP-seq, the other data and materials used in the analyses are available if required. All data generated and analysed during the current study are available from the corresponding author on reasonable request.
