# Supplementary material for: Single cell multi-omics of fibrotic kidney reveal epigenetic regulation of antioxidation and apoptosis within proximal tubule
Source: Cell Mol Life Sci. 2024 Jan 25;81(1):56. doi: 10.1007/s00018-024-05118-1 (PMC10811088; doi:10.1007/s00018-024-05118-1)
Supplement: Supplementary file 1 — Supplementary file1 (DOCX 3332 KB) [file 18_2024_5118_MOESM1_ESM.docx]

**Supplemental information**

**Cell-cell interaction within human and mouse fibrotic kidney**

In order to find cell-cell interaction during fibrosis, we show the overall signaling patterns of con and uuo sample, then we can see MK signaling pathway was activated in PT of uuod2 comparing with that of con. GRN signaling was both activated in IC and macrophage of uuo group comparing with those of con. Within endo, SEMA3 and CALCR signaling was activated in uuo group comparing with con group, when uuo goes to day 7 rather than day2, KIT signaling was also activated in endo cells(Figure S2B). Within podo, BMP, CALCR and TWEAK signaling activated in uuod2 group comparing with con, while increased BMP and TWEAK signaling in uuod2 disappear in uuod7. Interestingly, NRG, BMP and COMPLEMENT signaling within PT-inj were all enabled in uuo group comparing with con group. In LOH, CX3C signaling was increased in uuo compared with con. GDF and EDN signaling were respectively activated in PC and IC at 2 day after uuo. While at 7 day after uuo, besides EDN, KIT and GRN was also activated in IC, when at 2 day after uuo, GRN, CSF, COMPLEMENT, IFN-II and CX3C were activated in macrophage, in addition to IFN-II, other activated signaling persist until 7 day after uuo. Especially, OSM signaling was activated in neu of uuo group comparing with that of con. In myo, NT signaling was activated in uuod2, while in uuod7, OSM was activated (Figure S2A&B). Previous studies have found macrophage plays an important role in UUO development(1-3). So we use netAnalysis_dot function to see the interaction of macrophage with all cell types across con, uuod2, uuod7 and ruuo samples. And we found the Mif and Ccl4 secreted from macrophage respectively acting on (Cd74+Cd44) and Ccr5 of macrophage were activated in uuod2 samples comparing with con sample. The interaction between the Ccl6 and Ccr2 within macrophage were activated in uuod7 samples comparing with con sample. And Ccl9 and Ccl6 secreted from macrophage respectively acting on Ccr1 of neu were activated in uuo sample comparing with con(Figure S2D). The CCL, TGFb and PDGF signaling of all cell types across con, uuod2, uuod7 and ruuo samples were showed in VlnPlot. We can see obvious Ccr2 and Ccr5 within macrophage, Ccl3, Ccl4 and Ccr1 within neu and Ccl5 within T were all enabled in uuo samples compared with con samples(Figure S2E). When look into TGFb signaling pathway, more expression of Tgfbr2 within PT-Inj, Tgfbr1 within LOH and PC were observed(Figure S2F). When probe into PDGF signaling, Pdgfd within PT-Inj and Pdgfra within myo were activated in uuod7 compared with con kidneys(Figure S2G).The overall signaling patterns of Donor (Figure S2H) and CIN (Figure S2I) within human kidney were also visualized by Heatmap. Within podo, MK and PROS signaling were activated in CIN group comparing with donor, within endo, SEMA3 signaling was activated in CIN group comparing with donor. While IFN-II was activated in mac, neu, CD8 and NKT of CIN comparing with those of donor(Figure S2H&I).Then the TGFb (Figure S2J) and PDGF (Figure S2K) signaling of all cell types across Donor and CIN samples were showed in VlnPlot. Similar to mouse kidney, more expression of TGFBR1 within NKT, more expression of TGFBR2 within podo, LOH, T, CD8, NKT and more expression of TGFB1 within mac, T and NKT in CIN were observed(Figure S2J). When explore the PDGF signaling, PDGFC within PT and mac, PDGFRA within podo and PDGFD within NKT were activated in CIN compared with Donor(Figure S2K).

**DEGs were found for KEGG signaling enrichment by GSVA analysis**

In order to find the molecular mechanism underlying the fibrotic phenotype, differential expressed genes(DEGs) (adj p value<0.05) across each cell types between con and uuod7 or Donor and CIN was showed in Figure S3A and S3B, and the top 5 genes of average log2FC was marked by gene symbol(Figure S3A&B). Then DEGs within PT between con and uuod7 was analyzed by GSVA to enrich KEGG signaling pathway(Figure S3C), and DEGs within PT between donor and CIN was also analyzed by GSVA to enrich KEGG signaling pathway(Figure S3E). And the top signalings were showed by VlnPlot between con and uuod7(Figure S3D) or Donor and CIN(Figure S3F), we can see TGF-β, Wnt and Notch signaling was activated in uuod7 or CIN compared with con or donor, while glycolysis and TCA cycle signaling was downregulated in uuod7 or CIN(Figure S3D&F).

**Cell cluster specific TFs were found by SCENIC and ArchR**

To visualize the correspondence of all of our peak-to-gene links, we plot a peak-to-gene heatmap which contains two side-by-side heatmaps, left one for our scATAC-seq data and right one for our scRNA-seq data. The heatmap rows are clustered using k-means clustering based on the parameter k, which defaults to 25 as shown below. We can see the ATAC Z-Scores are consistent with the RNA Z-Scores(Figure S4A). Then we can also perform motif enrichment on our marker peaks across all cell types, the cell type specific TFs, which is showed in a heatmap, were analyzed by ArchR(Figure S4B). Then we overlay the TF deviation z-scores of each cell clusters on our UMAP embedding. To see how these TF deviation z-scores compare to the inferred gene expression via gene scores of the corresponding TF genes, we can overlay the linked gene expression for each of these TFs on the UMAP embedding(Figure S4C). And the cell type specific TFs within mouse and human kidney, which is respectively showed in the Figure S4D and Figure S4F, were analyzed by SCENIC. And the representative cell type TFs within mouse(Figure S4E) and human kidney(Figure S4G) were displayed in FeaturePlot.

**Subclusters of PT and myo and trajectory analysis of PT subclusters across mouse and human**

In order to investigate the mechanism underlying the maladaptive repair of PT cells and its role in promoting kidney fibrosis, we selected PT and myo to do subgroup analyses, within fibrotic mouse kidney, we subclustered PT and myo into 12 clusters, they are Kap hi-PT, Spp2 hi-PT, Fbp1 hi-PT, Miox hi-PT, Normal PT S1, Normal PT S2/3, FR-PT, injured PT S1/2, injured PT S3, inflammatory PT, pericyte and myo(Figure S5A). The markers to define these clusters were showed in DotPlot(Figure S5B). In order to find consistent phenomena, we also subclustered PT and myo within fibrotic human kidney, and there are 6 cell clusters including Normal PT, NNMT hi-PT, AKR1C1 hi-PT, MIOX hi-PT, FR-PT and myo(Figure S5E). The markers to define these clusters were showed in Figure S5F by DotPlot. In human kidney, we can see decreased cell proportion of AKR1C1 and NNMT hi-PT and increased cell proportion of FR-PT and myo in CIN group comparing with that in donor group(Figure S5J). While in mouse kidney, an increased cell proportion of FR-PT was observed in uuo group comparing with con group(Figure S5I). As we know, PT can transform into myo, then we make use of our snATAC-seq data to do the trajectory analysis by using ArchR R packages, then we can see PT transform into myo through pseudotime analysis(Figure S6A) and the change of TFs and its RNA expression was showed by heatmap(Figure S6B). The trajectory analysis of mouse PT and myo was showed in Figure S6C, and the split view of the trajectory analysis by various samples were also showed in Figure S6D. We also show the representative transcription factors and variable genes change over the process of mouse PT transforming into myo (Figure S6E). The trajectory analysis of human PT and myo was showed in Figure S6G, and the split view of the trajectory analysis by Donor and CIN were also showed(Figure S6H). Similarly, we show the representative transcription factors and variable genes change over the process of human PT transforming into myo (Figure S6I).

**
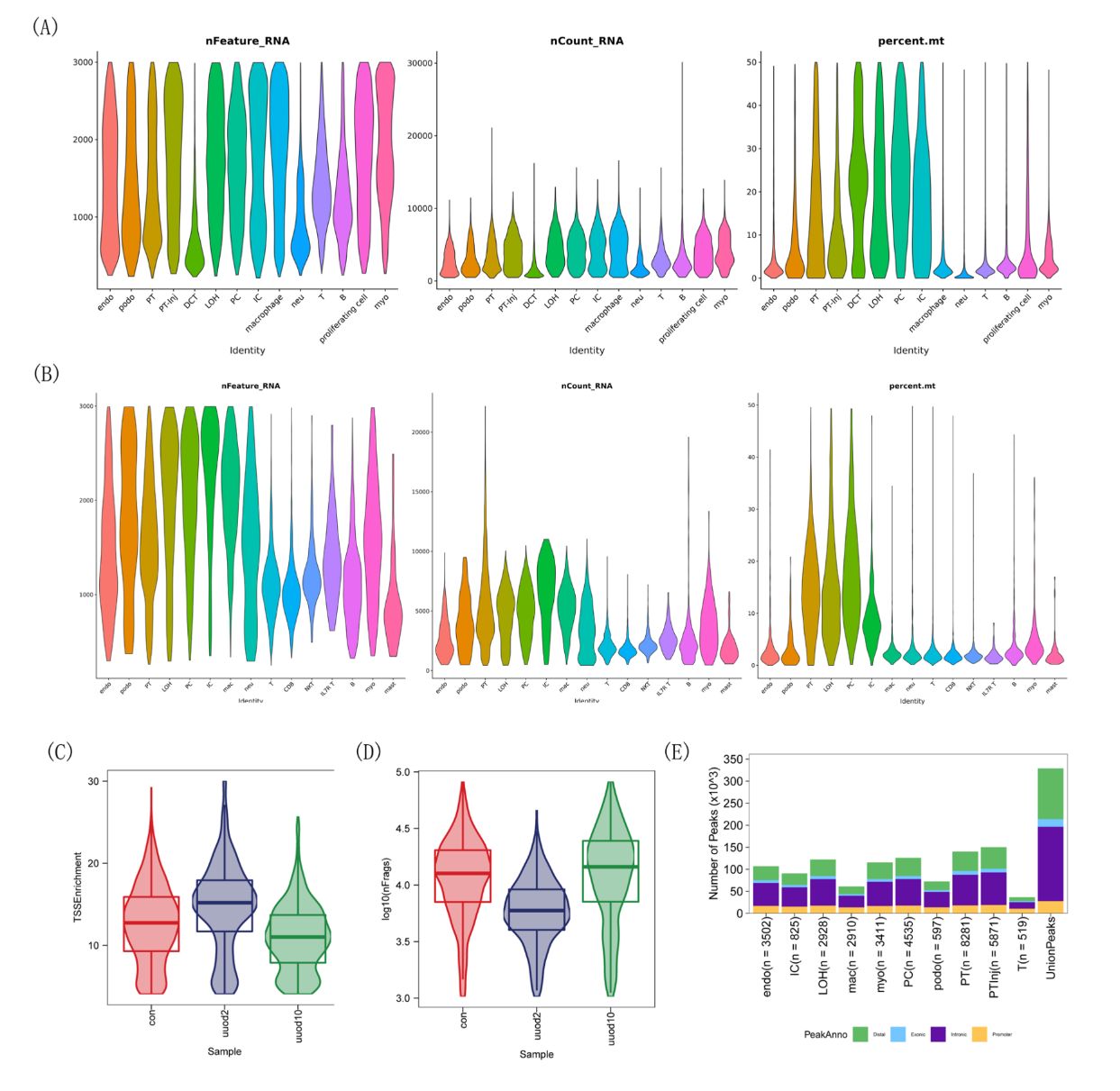
**

**Figure S1. Quality control of scRNA-seq and snATAC-seq.** **(A)**The cells within mouse kidney, which were less than 50% mitochondria transcripts, with more than 200 and less than 3000 genes were retained. And the nFeature, nCount, percent.mt of retained cells were showed by VlnPlot. **(B)** The cells within human kidney, which were less than 50% mitochondria transcripts, with more than 200 and less than 3000 genes were retained. And the nFeature, nCount, percent.mt of retained cells were showed by VlnPlot. The TSS enrichment**(C)** and log10(nFrags)**(D)** of snATAC-seq data containing con, uuod2 and uuod10 were showed by VlnPlot. **(E)**The number of Distal, exonic, intronic and promoter across all cell types was summarized by a bar chart.


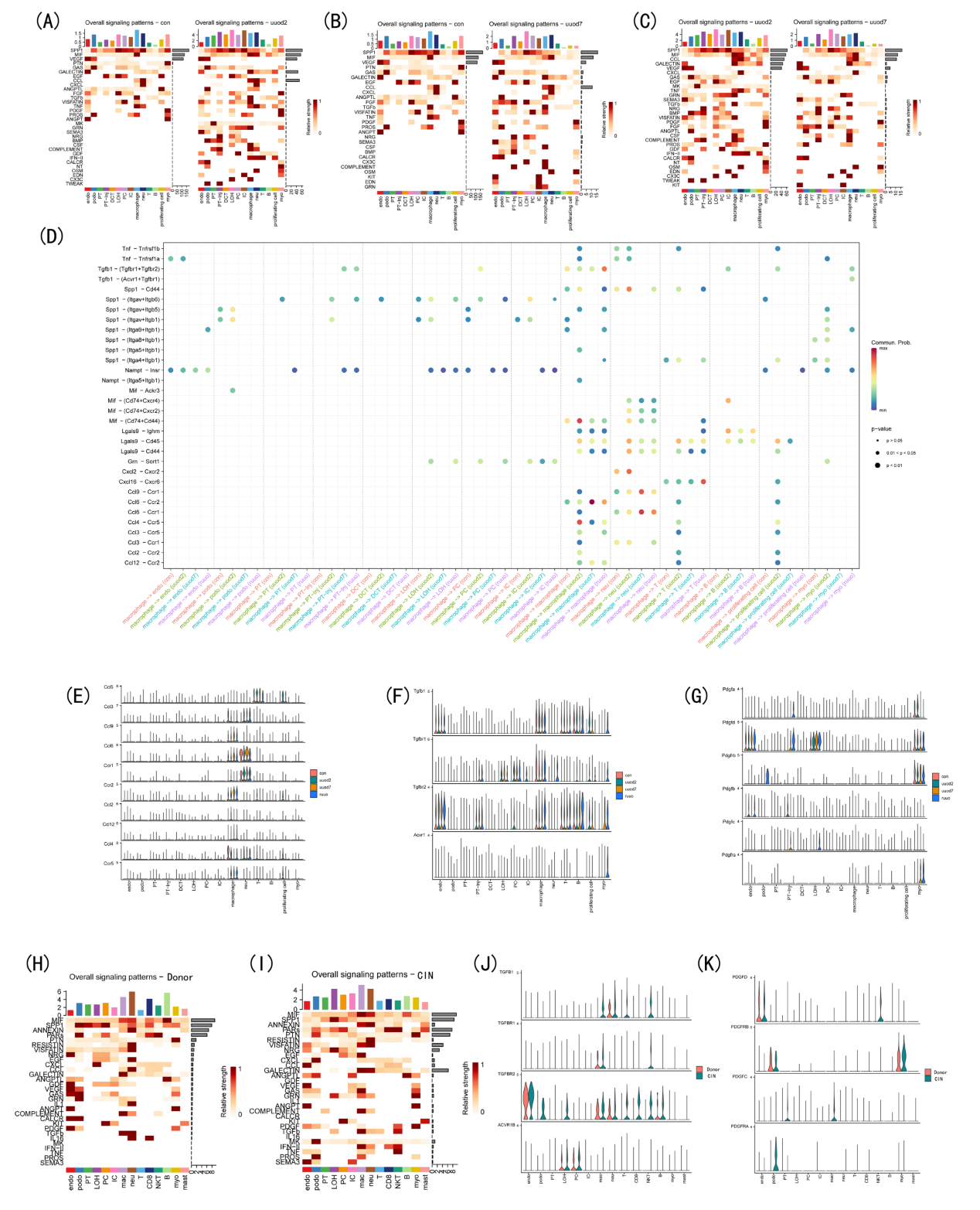


**Figure S2.** **Cell-cell interaction within human and mouse fibrotic kidney.** **(A)**The overall signaling patterns of con vs uuod2 within mouse kidney were visualized by Heatmap**. (B)** The overall signaling patterns of con vs uuod7 within mouse kidney were visualized by Heatmap. **(C)** The overall signaling patterns of uuod2 vs uuod7 within mouse kidney were visualized by Heatmap. **(D)** Previous studies have found macrophage plays an important role in UUO development. So we use netAnalysis_dot function to see the interaction of macrophage with all cell types across con, uuod2, uuod7 and ruuo samples. And we found Ccl-Ccr signalings were activated in uuo samples comparing with con sample. The CCL**(E)**, TGFb **(F)** and PDGF **(G)** signaling of all cell types across con, uuod2, uuod7 and ruuo samples were showed in VlnPlot. The overall signaling patterns of Donor **(H)** and CIN **(I)** within human kidneys were visualized by Heatmap**.** The TGFb **(J)** and PDGF **(K)** signaling of all cell types across Donor and CIN samples were showed in VlnPlot**.**


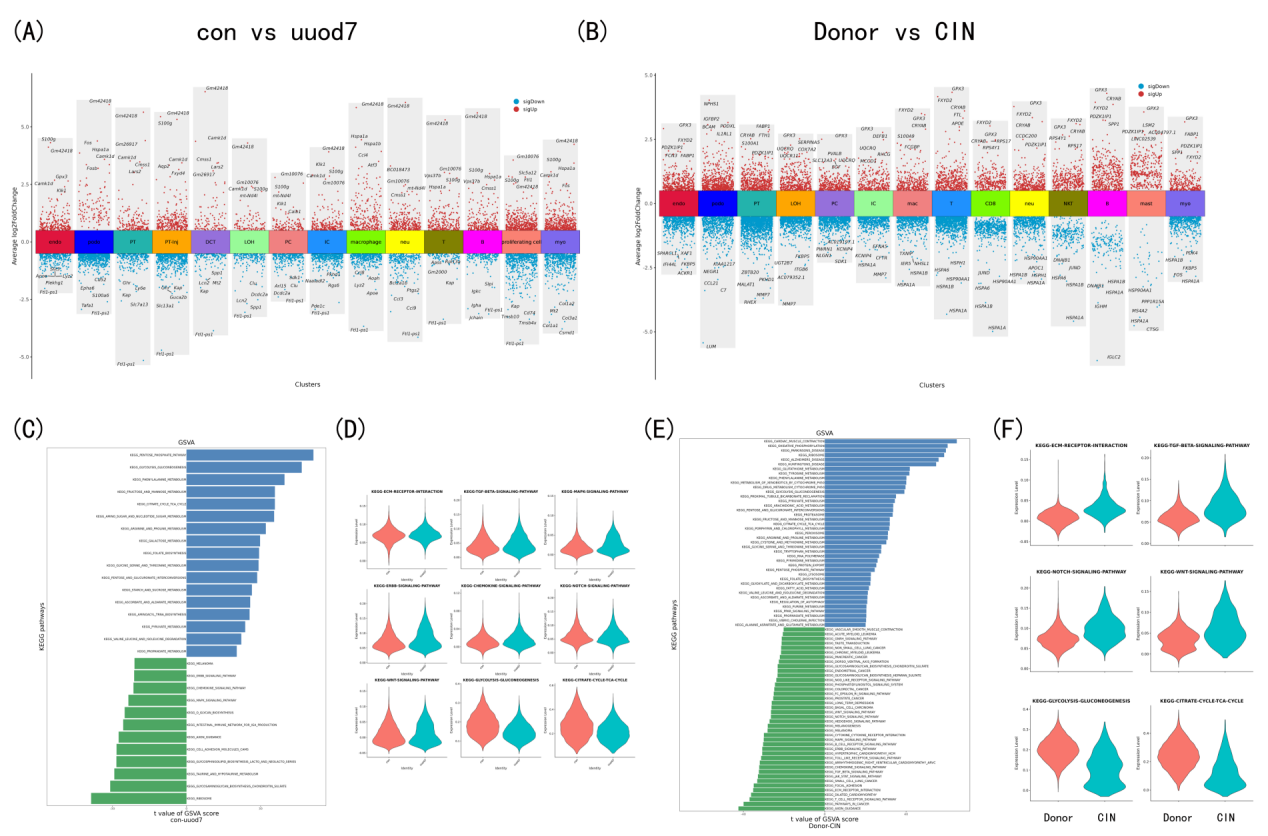


**Fig. S3. Differential expressed genes across all cell types and GSVA analysis within PT between fibrotic and con kidney.** **(A)**The differential expressed genes across all cell types between con and uuod7 were showed by Volcano Plot, and the top 5 genes ranked by average log2FC were marked by gene symbol. **(B)** The differential expressed genes across all cell types between donor and CIN were showed by Volcano Plot, and the top 5 genes ranked by average log2FC were marked by gene symbol. **(C)** The GSVA pathway activity, based on KEGG of the MSigDB database, in the con group compared to uuod7 was visualized by a bar plot. **(D)** The representative KEGG signaling pathway from figC were visualized as VlnPlot. **(E)** The GSVA pathway activity, based on KEGG of the MSigDB database, in the donor group compared to CIN group was visualized by a bar plot. **(F)** The representative KEGG signaling pathway from figE were visualized as VlnPlot.


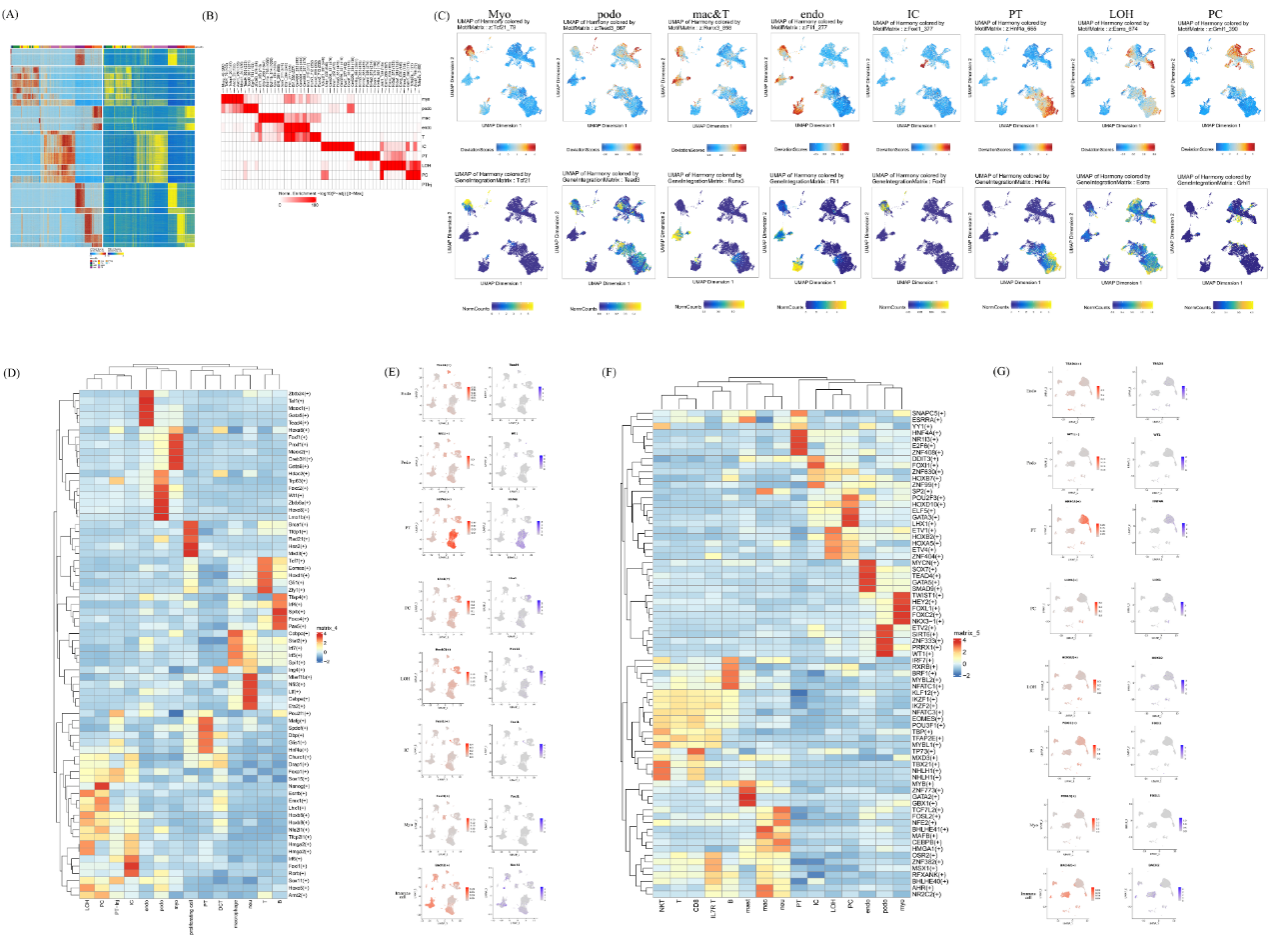


**Figure S4.Cell clusters specific TFs found by ArchR and SCENIC.** **(A)**A peak-to-gene heatmap which contains two side-by-side heatmaps, left one for our scATAC-seq data and right one for our scRNA-seq data. The heatmap rows are clustered using k-means clustering based on the parameter k, which defaults to 25 as shown below. **(B)** The cell type specific TFs showed in a heatmap were analyzed by ArchR. **(C)** The TF deviation z-scores of each cell clusters and the linked gene expression for each of these TFs on our UMAP embedding. The cell type specific TFs within mouse and human kidney respectively showed in the figD and figF were analyzed by SCENIC. And the representative cell type TFs within mouse **(E)** and human **(G)** kidney were displayed in FeaturePlot.

**
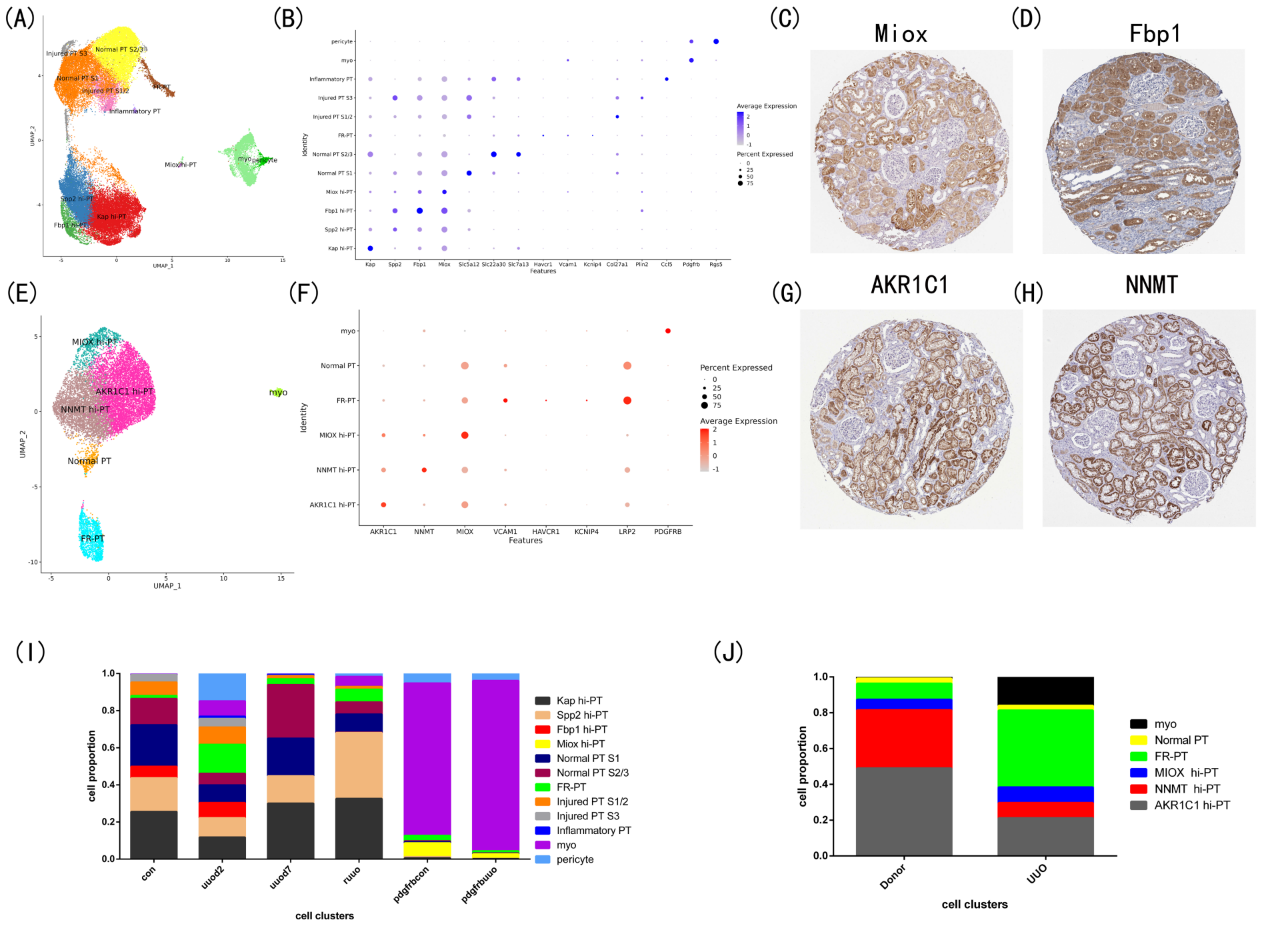
Figure S5. Subclusters of PT and myo. (A)**The subclusters of PT and myo from mouse scRNA-seq data were visualized in UMAP, and the cell annotation was marked in the Plot. **(B)**The markers to define the cell names from figA were showed by DotPlot. The IHC staining of MIOX**(C),** FBP1**(D),** AKR1C1**(G)** and NNMT**(H),** from The Human Protein Altas(39)(https://www.proteinatlas.org/), were showed. **(E)** The subclusters of PT and myo from human scRNA-seq data were visualized in UMAP, and the cell annotation was marked in the Plot. **(F)** The markers to define the cell names from figE were showed by DotPlot. **(I)** The cell proportion in PT and myo subclusters from mouse scRNA-seq data. **(J)** The cell proportion in PT and myo subclusters from human scRNA-seq data.

**
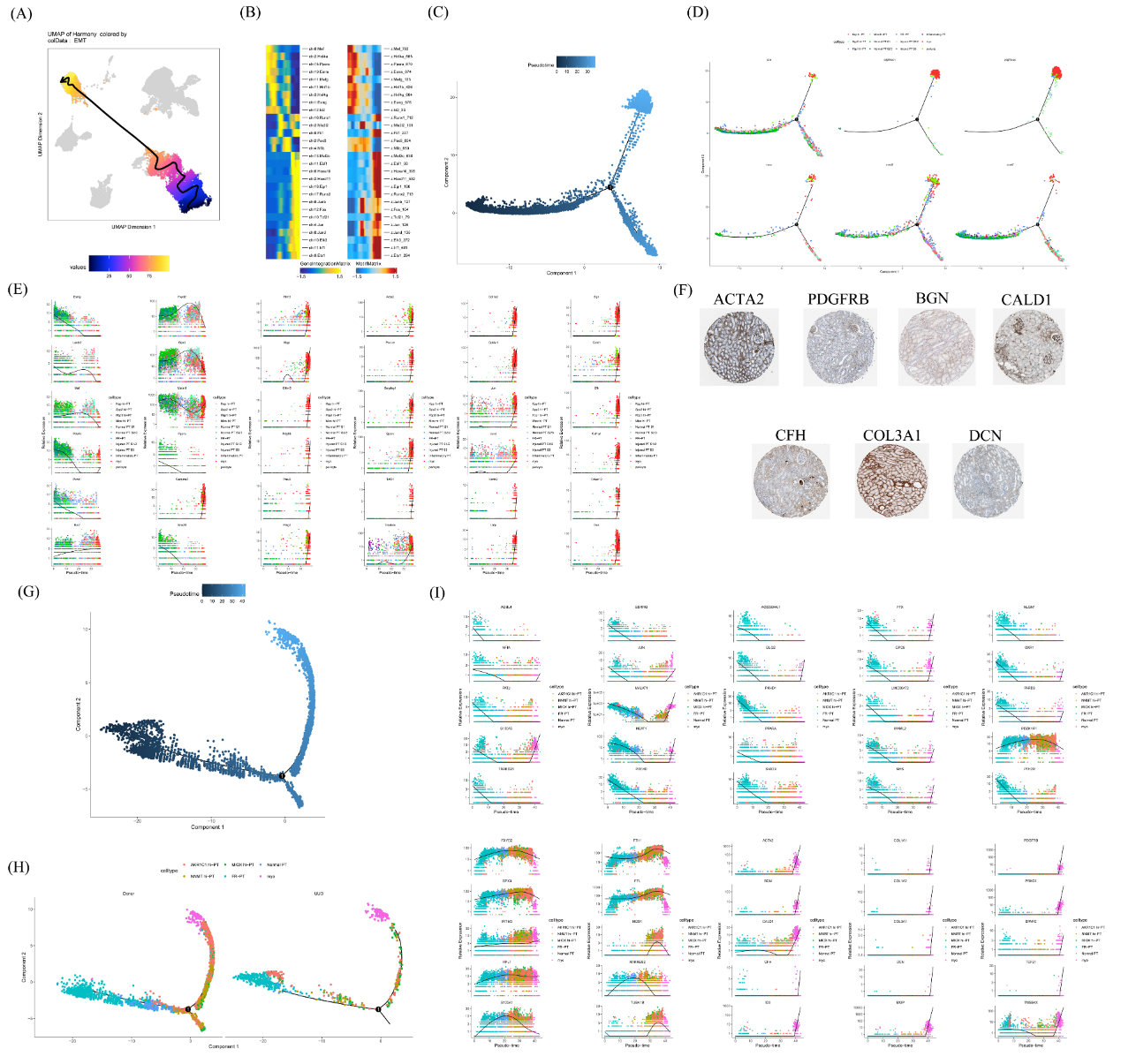
**

**Figure S6.** **Trajectory analysis of PT and myo clusters within mouse and human kidney.** **(A)**The pseudotime analysis of PT and myo clusters in snATAC-seq data was visualized by UMAP Plot. **(B)**When PT transit to myo, the alteration patterns of gene integration and motif matrix of transcription factors were showed by Heatmap. **(C)** The pseudotime analysis of PT and myo clusters in mouse scRNA-seq data was visualized by plot_cell_trajectory function. **(D)**The split view of PT and myo clusters from each mouse samples was visualized by facet_wrap function of plot_cell_trajectory. **(E)** In mouse kidney, the representative transcription factors and variable genes during PT transition to myo were showed by plot_genes_in_pseudotime function**. (F)**The representative IHC staining of myo markers, from The Human Protein Altas(4)(https://www.proteinatlas.org/), were showed. **(G)** The pseudotime analysis of PT and myo clusters in human scRNA-seq data was visualized by plot_cell_trajectory function. (H) The split view of PT and myo clusters from each human samples was visualized by facet_wrap function of plot_cell_trajectory. (I) In human kidney, the representative transcription factors and variable genes during PT transition to myo were showed by plot_genes_in_pseudotime function.

**References**

1. Wu Q, Sun S, Wei L, Liu M, Liu H, Liu T, et al. Twist1 regulates macrophage plasticity to promote renal fibrosis through galectin-3. *Cellular and molecular life sciences : CMLS.* 2022;79(3):137.

2. Chen J, Tang Y, Zhong Y, Wei B, Huang X-R, Tang PM-K, et al. P2Y12 inhibitor clopidogrel inhibits renal fibrosis by blocking macrophage-to-myofibroblast transition. *Molecular Therapy.* 2022;30(9):3017-33.

3. Fu H, Gu Y-H, Tan J, Yang Y-N, and Wang G-H. CircACTR2 in macrophages promotes renal fibrosis by activating macrophage inflammation and epithelial–mesenchymal transition of renal tubular epithelial cells. *Cellular and Molecular Life Sciences.* 2022;79(5):253.

4. Uhlén M, Fagerberg L, Hallström BM, Lindskog C, Oksvold P, Mardinoglu A, et al. Tissue-based map of the human proteome. *Science (New York, NY).* 2015;347(6220):1260419.
